# Lung water density is increased in patients at risk of heart failure and is largely independent of conventional cardiovascular magnetic resonance measures

**DOI:** 10.1093/ehjimp/qyae089

**Published:** 2024-08-27

**Authors:** Nithin R Iyer, Jennifer A Bryant, Thu-Thao Le, Justin G Grenier, Richard B Thompson, Calvin W L Chin, Martin Ugander

**Affiliations:** Kolling Institute, Royal North Shore Hospital, The University of Sydney, St Leonards, NSW, Australia; Department of Cardiology, National Heart Centre Singapore, Singapore, Singapore; Department of Cardiology, National Heart Centre Singapore, Singapore, Singapore; Department of Cardiology, National Heart Centre Singapore, Singapore, Singapore; Cardiovascular Sciences ACP, Duke NUS Medical School, Singapore, Singapore; Department of Radiology and Diagnostic Imaging, University of Alberta, Edmonton, Canada; Department of Radiology and Diagnostic Imaging, University of Alberta, Edmonton, Canada; Department of Cardiology, National Heart Centre Singapore, Singapore, Singapore; Cardiovascular Sciences ACP, Duke NUS Medical School, Singapore, Singapore; Kolling Institute, Royal North Shore Hospital, The University of Sydney, St Leonards, NSW, Australia; Department of Clinical Physiology, NKS C8:27, Karolinska University Hospital, Karolinska Institutet, SE-17176 Stockholm, Sweden

## Abstract

**Aims:**

Non-invasive methods to quantify pulmonary congestion are lacking in clinical practice. Cardiovascular magnetic resonance (CMR) lung water density (LWD) mapping is accurate and reproducible and has prognostic value. However, it is not known whether LWD is associated with routinely acquired CMR parameters.

**Methods and results:**

This was an observational cohort including healthy controls and patients at risk of heart failure. LWD was measured using CMR with a free-breathing short echo time 3D Cartesian gradient-echo sequence with a respiratory navigator at 1.5 T. Associations were assessed between LWD, lung water volume and cardiac volumes, left ventricular (LV) mass and function, myocardial native T1, and extracellular volume fraction. In patients at risk for heart failure (*n* = 155), LWD was greater than in healthy controls (*n* = 15) (30.4 ± 5.0 vs. 27.2 ± 4.3%, *P* = 0.02). Using receiver operating characteristic analysis, the optimal cut-off for LWD was 27.6% to detect at-risk patients (sensitivity 72%, specificity 73%, positive likelihood ratio 2.7, and inverse negative likelihood ratio 2.6). LWD was univariably associated with body mass index (BMI), hypertension, right atrial area, and LV mass. In multivariable linear regression, only BMI remained associated with LWD (*R*^2^ = 0.32, *P* < 0.001).

**Conclusion:**

LWD is increased in patients at risk for heart failure compared with controls and is only weakly explained by conventional CMR measures. LWD provides diagnostic information that is largely independent of conventional CMR measures.

## Graphical Abstract

**Graphical Abstract:**
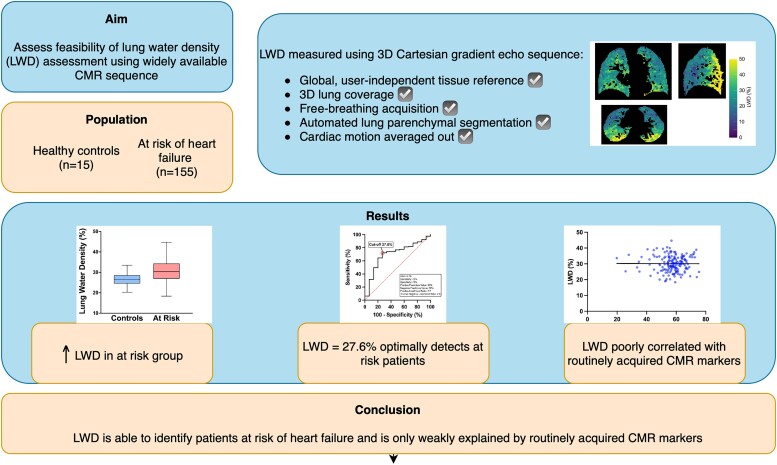

## Introduction

Pulmonary oedema is the accumulation of extravascular fluid in the lung parenchyma. Cardiogenic pulmonary oedema occurs in heart failure due to an increase in hydrostatic pressure in the pulmonary capillaries resulting from increased left atrial (LA) pressure and is associated with a poor long-term prognosis.^1–3^ Current methods to assess pulmonary oedema are either invasive (transpulmonary thermodilution) or semi-quantitative (chest X-ray and lung ultrasound) or require the use of ionizing radiation [nuclear imaging and computed tomography (CT)].

Cardiovascular magnetic resonance (CMR) imaging is a non-invasive imaging modality that is well suited to quantify lung water content without the need for ionizing radiation and has shown good correlation with gravimetric lung weight in animal models.^4^ In human studies, estimated lung water density (LWD) in a heart failure cohort measured with the widely available single-shot fast spin-echo (HASTE) pulse sequence was shown to be correlated with invasively measured left-sided filling pressures and predictive of cardiovascular outcomes.^5^ However, this method has a number of limitations, including (i) lack of a robust reference signal, (ii) incomplete spatial coverage of the lungs, and (ii) time-consuming methodology involving 30-min supine positioning before CMR. A subsequent retrospective study, using the same sequence, demonstrated the feasibility of LWD measurement without the need for lengthy supine positioning prior to CMR and used a single HASTE sequence acquisition at end expiration.^6^ LWD was once again found to predict cardiovascular outcomes in patients with heart failure.

More recently, custom non-Cartesian gradient-echo pulse sequences with short echo times have been shown to provide 3D LWD acquisitions in a single breath hold or respiratory navigator free-breathing acquisitions.^2,7–9^ These methods have addressed practical challenges that must be overcome for accurate estimation of LWD including the low signal-to-noise ratio due to low proton density in the lungs, rapid T2* signal decay, large required field of view and 3D spatial coverage, respiratory motion, the need for a reliable signal reference, and magnetic resonance imaging (MRI) signal normalization.^2^ However, these custom methods require specialized MRI hardware including either low field-strength (0.55 T) MRI scanners or high gradient slew rates (175 T/m/s), which may not be readily available.

A standard 3D Cartesian gradient-echo pulse sequence has recently been optimized for LWD quantification in routinely available 1.5 T clinical MRI scanners. Modifications to the pulse sequence, including a shorter radiofrequency pulse (10 µs) and optimized gradients, result in an acceptable 3.75 mm isotropic resolution in patient-friendly (<10 s) acquisition times. The sequence offers 3D full torso coverage, a built-in respiratory navigator, and improved background correction and automated lung parenchymal segmentation. The sequence benefits from the advantages of Cartesian sampling over non-Cartesian methods, including better imaging efficiency, shorter reconstruction times, and being less prone to imaging artefacts.^10^

The goals of the current study, therefore, were to (i) assess feasibility of LWD measurement in healthy controls and patients at risk of heart failure using a 3D Cartesian gradient-echo approach and (ii) evaluate the association between LWD and routinely acquired CMR markers including cardiac volumes, mass, function, and myocardial extracellular volume (ECV) fraction.

## Methods

### Study population

This observational study cohort consisted of (i) subjects prospectively and serially recruited at the time of clinical CMR at the National Heart Centre Singapore from May 2021 through March 2022 and (ii) patients with hypertension undergoing CMR as part of ongoing clinical trials at the centre. Asymptomatic controls without known cardiac disease were recruited. Patients were included if they were aged ≥21 years and able to receive intravenous gadolinium-based contrast agents and had no contraindications to CMR. Exclusion criteria included a documented diagnosis of heart failure, missing relevant demographic data, or un-interpretable imaging. Patients were retrospectively classified as at risk of heart failure if they had a history of coronary artery disease, diabetes mellitus or hypertension, or clinical history or CMR evidence of cardiomyopathy (ischaemic, hypertrophic, dilated, and amyloidosis). Ethics approval was obtained from the SingHealth Centralized Institutional Review Board, and all participants provided written informed consent. The study was conducted in accordance with the principles of the Declaration of Helsinki.

### CMR image acquisition

All patients underwent standardized CMR (Siemens Aera 1.5 T; Siemens Healthineers, Erlangen, Germany). The study design did not include the administration of a gadolinium-based contrast agent for the healthy volunteer group in order to minimize associated risks. Balanced steady-state free precession cine images were acquired in the standard long-axis views and a short-axis stack from base to apex, as described previously.^11^ Late gadolinium enhancement (LGE) images were acquired at 8 min after 0.1 mmol/kg of gadobutrol (Gadovist®, Bayer Pharma AG, Germany) with a phase-sensitive inversion recovery fast gradient-echo imaging sequence. The inversion time for optimal myocardial nulling was selected from an inversion time scout sequence. T1 maps were acquired at the basal and mid-ventricular short-axis levels, pre- .and 15-min post-contrast with modified Look-Locker inversion recovery (MOLLI) 5 s(3 s)3 s and 4 s(1 s)3 s(1 s)2 s acquisition schemes, respectively. LWD was measured with a free-breathing short echo 3D Cartesian gradient-echo sequence with a respiratory navigator. Typical imaging parameters included a matrix size of 96 × 132 × 60 with a 360 mm × 500 mm × 300 mm field of view in the head to foot, right to left, and chest to back directions, respectively, 2000 Hz/pixel readout bandwidth, echo time = 0.43 ms, repetition time = 1.5 ms, and flip angle 1°. The low flip angle and short echo time were selected to ensure primarily water density image contrast with minimal T1 and T2* weighting.^2^ All images were interpolated to 2.5 mm isotropic resolution. Complete acquisitions were repeated seven times over 84 s of tidal respiration with the insertion of a centre of k-space navigator acquisition every 72 lines of k-space (every 108 ms).

### CMR analysis

Image analysis was performed at the National Heart Research Institute Singapore CMR Core Laboratory using CVI42 software (Circle Cardiovascular Imaging, Calgary, Canada) by trained imaging fellows who were blinded to the clinical data. Cardiac volumes, mass, and ejection fraction were analysed according to standardized protocols.^12,13^ Left ventricular (LV) volumes and mass data were indexed to body surface area (BSA). The presence of LGE was assessed qualitatively by two readers according to the recommendations by the Society of CMR.^14^ Myocardial native T1 and ECV fraction were measured while excluding regions of focal LGE, as described previously.^11^ Myocardial strain was analysed in the cine images using the tissue tracking plugin.^13^ Global LV wall thickness, an early marker of LV hypertrophy, was defined as $0.05+1.60\cdot \mathrm{LV}M^{0.84}\cdot \mathrm{LVED}V^{-0.49}$, where LVM is LV mass in grams and LVEDV is LV end-diastolic volume in millilitres.^15^

### Lung water analysis

The centre of k-space respiratory navigator was used to reconstruct a single 3D lung data set at the functional residual capacity respiratory phase (minimum lung volume).^2^ Lung water images were processed offline using a custom program in MATLAB (The MathWorks, Natick, MA, USA).^2^ A spatial normalization scheme was applied to eliminate surface coil shading, as previously described.^2^ Briefly, all solid tissues surrounding the lungs were fit with a low spatial frequency normalization map that was interpolated over the full field. 3D images were then corrected by division with the normalization map to yield lung voxel intensity in units of relative LWD. The lung parenchyma, excluding large vessels, was identified with a region-growing algorithm.^2^ Lung volume was calculated as the total volume of the voxels in the lung mask. Average LWD was calculated as the mean LWD value from all lung parenchyma voxels. Water volumes in each voxel were calculated as the LWD value multiplied by the voxel volume and the total lung water volume (LWV) as the sum of these volumes over all lung voxels. The LWV index (LWVi) was calculated as the LWV indexed to BSA.

### Statistics

Normality was assessed for continuous variables using the Shapiro–Wilk test. Normally distributed data are presented as mean ± standard deviation. Non-normally distributed data are presented as median [interquartile range]. Comparisons were performed for continuous variables using the parametric Student *t*-test or the non-parametric Mann–Whitney *U* test. Categorical variables are presented as number (percentage) and compared using the *χ*^2^ test. Pearson’s rank order test was performed to detect significant correlations between LWD, LWV, and routine CMR markers including cardiac volumes, LV mass and ejection fraction, myocardial native T1, and ECV. Multiple linear regression was performed to assess the relationship between LWD, LWV, and routinely acquired CMR markers, adjusting for relevant clinical confounders including age, sex, BSA, body mass index (BMI), hypertension, diabetes, coronary artery disease, New York Heart Association class, and systolic blood pressure (BP). Covariates with *P* < 0.05 in univariable analyses were entered into the multivariable linear regression model to identify independent predictor variables, using forward stepwise selection with a probability to remove threshold set to ≥0.1. Multicollinearity was assessed with a correlation matrix for predictor variables, using a threshold coefficient value of 0.7. Receiver operating characteristic analysis with Youden’s index was used on the entire cohort to identify an optimal LWD cut-off for discriminating between the control and at-risk groups. Diagnostic performance was described using sensitivity, specificity, positive and negative predictive values, and the disease prevalence-independent positive and inverse negative likelihood ratios. Statistical analyses were performed using SPSS Version 28 (Statistical Package for the Social Sciences, International Business Machines, Inc., Armonk, NY, USA) and GraphPad Prism 9.4.1 (GraphPad Software, Inc., San Diego, CA, USA). A two-sided *P*-value of <0.05 was considered as statistically significant.

## Results

**Figure 1:**
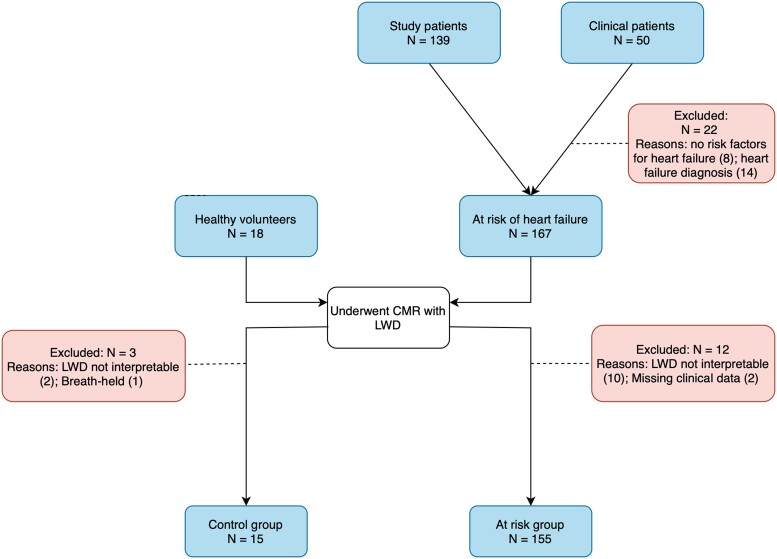
Flow chart of patient inclusion.

A flowchart of patient selection is shown in *Figure 1*. Out of the 50 patients undergoing clinically indicated CMR recruited for the study, 22 were excluded due to the absence of risk factors for heart failure or due to a pre-existing diagnosis of heart failure. This group of 28 patients, in conjunction with 139 patients with at least one risk factor for heart failure enrolled in ongoing clinical trials at our centre, underwent CMR including LWD. From these 167 patients, 12 patients had missing clinical data or un-interpretable LWD data and were excluded. Out of the 18 healthy volunteers who underwent CMR with LWD, 3 were excluded due to un-interpretable LWD data or breath-held acquisition. In total, 170 subjects (healthy controls, *n* = 15; at risk of heart failure, *n* = 155) were included in the study cohort (age range: 24–79 years; males: 69%).

### Baseline characteristics

**Figure 2:**
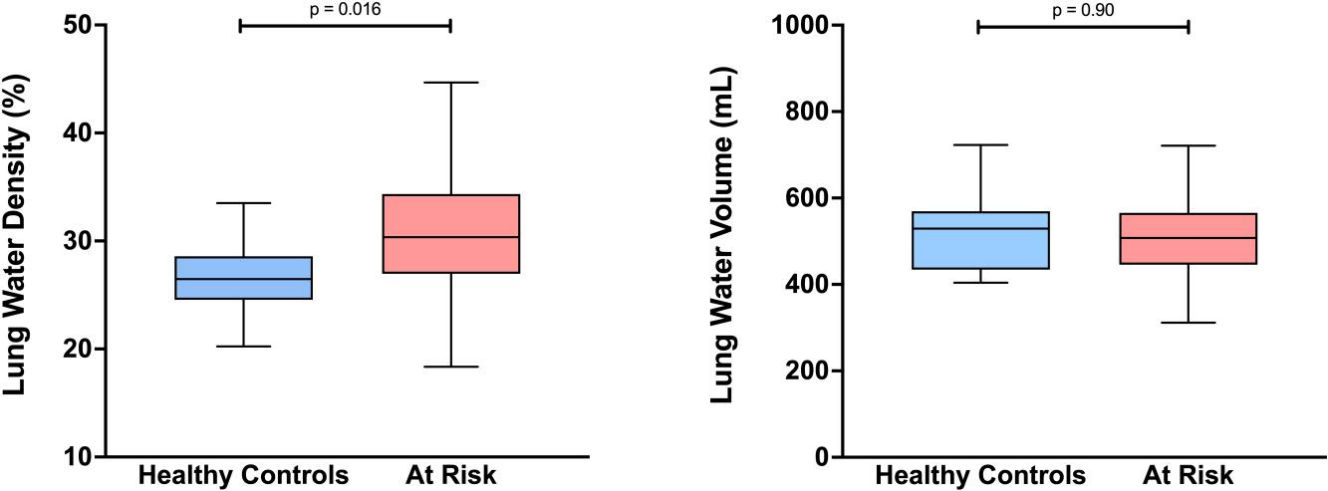
LWD and LWV according to clinical group: LWD was higher in patients at risk of heart failure compared with healthy controls (*A*); LWV did not differ between healthy controls and patients at risk of heart failure (*B*). Whiskers were plotted using the Tukey method. Outliers are not shown for clarity.

The baseline clinical characteristics are shown in *Table 1*. Compared with healthy controls, patients at risk of heart failure were older, had higher systolic BP, and had higher BMI. At CMR, the at-risk group had higher right atrial (RA) area, LA volume, LV mass and global LV wall thickness (GT), worse global longitudinal strain (GLS), and higher LWD (*Figure 2*). LWV did not differ between the two groups. LWVi and lung volume trended lower in the at-risk group, but these results were not statistically significant.

**Table 1:** Baseline clinical and CMR characteristics of the cohort

	Healthy controls (*n* = 15)	At risk of heart failure (*n* = 155)	*P*-value
Clinical			
Age, years	48 [38–55]	60 [50–65]	<0.001
Male sex, *n* (%)	8 (53)	110 (71)	0.16
Systolic blood pressure, mmHg	131 ± 19	142 ± 18	0.006
BSA, m^2^	1.73 ± 0.25	1.86 ± 0.23	0.006
BMI, kg/m^2^	21.6 [19.5–26.5]	27.0 [23.8–30.1]	<0.001
Co-morbidities, *n* (%)			
Hypertension	0 (0)	143 (92)	
Diabetes	0 (0)	62 (40)	
Coronary artery disease	0 (0)	37 (24)	
NYHA functional class, *n* (%)			
Class I	15 (100)	117 (89)	
Class II	0 (0)	15 (11)	
CMR markers			
RA area, m^2^	12 [11–13]	20 [16–23]	<0.001
LA volume, mL	70 [64–81]	86 [73–104]	0.013
LA volume indexed to BSA, mL/m^2^	42 [38–47]	46 [39–54]	0.043
LV EDV, mL	131 [120–144]	134 [112–158]	0.80
RV EDV, mL	142 [122–153]	134 [110–156]	0.28
LV mass, g	76 [69–98]	97 [80–117]	0.006
GT, mm	5.8 [5.4–6.6]	6.8 [6.0–7.5]	0.001
LV ejection fraction, %	57 [56–61]	58 [54–63]	0.84
GLS, %	−18.2 ± 1.7	−15.6 ± 3.3	0.004
LGE type			
Nil, *n* (%)	9 (100)	88 (57)	
Non-ischaemic, *n* (%)	0 (0)	36 (23)	
Ischaemic, *n* (%)	0 (0)	29 (19)	
Both, *n* (%)	0 (0)	2 (1)	
Native T1, ms		1022 [1008–1038]	
ECV, %		25.6 [23.8–27.2]	
Lung volume, mL	1861 [1604–2405]	1708 [1416–2037]	0.08
LWD, %	27.2 ± 4.3	30.4 ± 5.0	0.02
LWV, mL	521 ± 94	518 ± 98	0.90
LWVi, mL/m^2^	303 ± 42	279 ± 47	0.058

Values are given as median [interquartile range], mean ± SD, or number (percentage).

BSA, body surface area; NYHA, New York Heart Association; RA, right atrial; LA, left atrial; LV, left ventricular; EDV, end-diastolic volume; RV, right ventricular; GT, global LV wall thickness; GLS, global longitudinal strain; LGE, late gadolinium enhancement; ECV, extracellular volume; LWD, lung water density; LWV, lung water volume; LWVi, lung water volume indexed to body surface area.

### Receiver operating characteristic analysis

**Figure 3:**
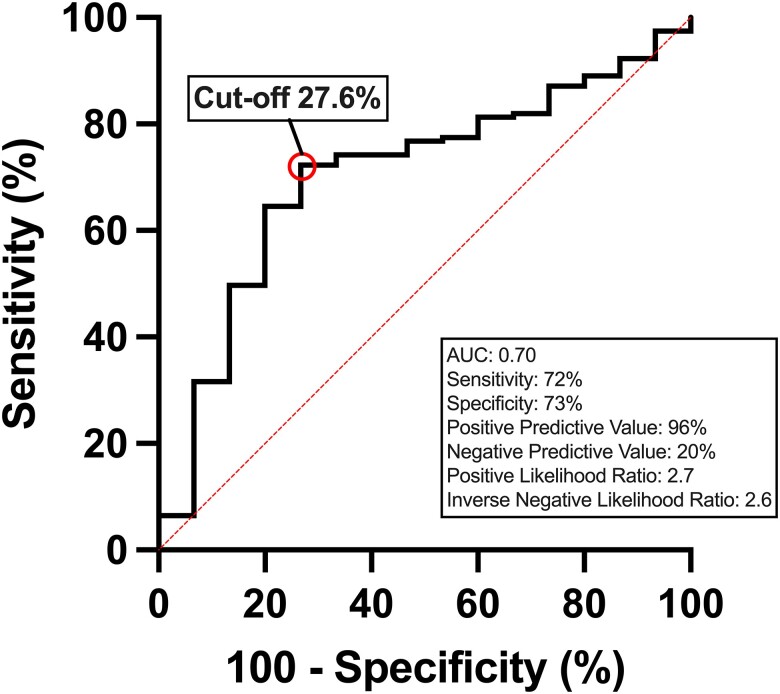
Receiver operating characteristic curve and corresponding AUC describing the diagnostic performance of LWD to detect patients at risk of heart failure.

Using receiver operating characteristic analysis, the area under the curve (AUC) was 0.70 [95% confidence interval (CI) 0.58–0.83] for LWD to differentiate between healthy controls and patients at risk of heart failure (*Figure 3*). The optimal cut-off point for LWD was 27.6% to detect at-risk patients with sensitivity 72%, specificity 73%, positive predictive value 96%, negative predictive value 20%, positive likelihood ratio 2.7, and inverse negative likelihood ratio 2.6. For LWV, the AUC was 0.49 (95% CI 0.34–0.65), indicating no ability to discriminate between healthy controls and patients at risk of heart failure.

### Variables associated with LWD

**Figure 4:**
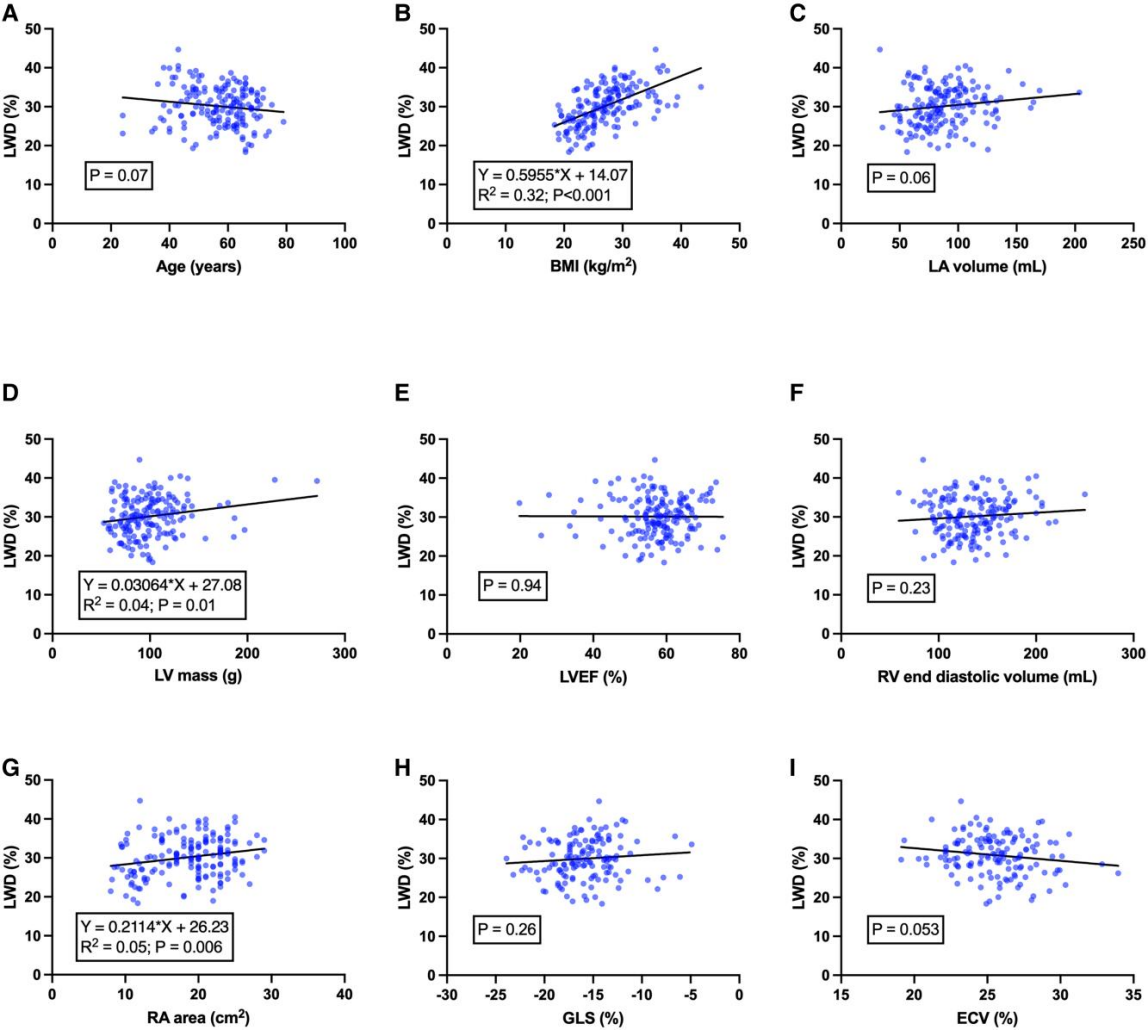
Scatter plots showing univariable relationships between LWD and clinical or routinely acquired CMR markers. LWD was significantly associated with BMI (*B*), LV mass (*D*), and RA area (*G*). The line of best fit is shown for each analysis.

**Figure 5:**
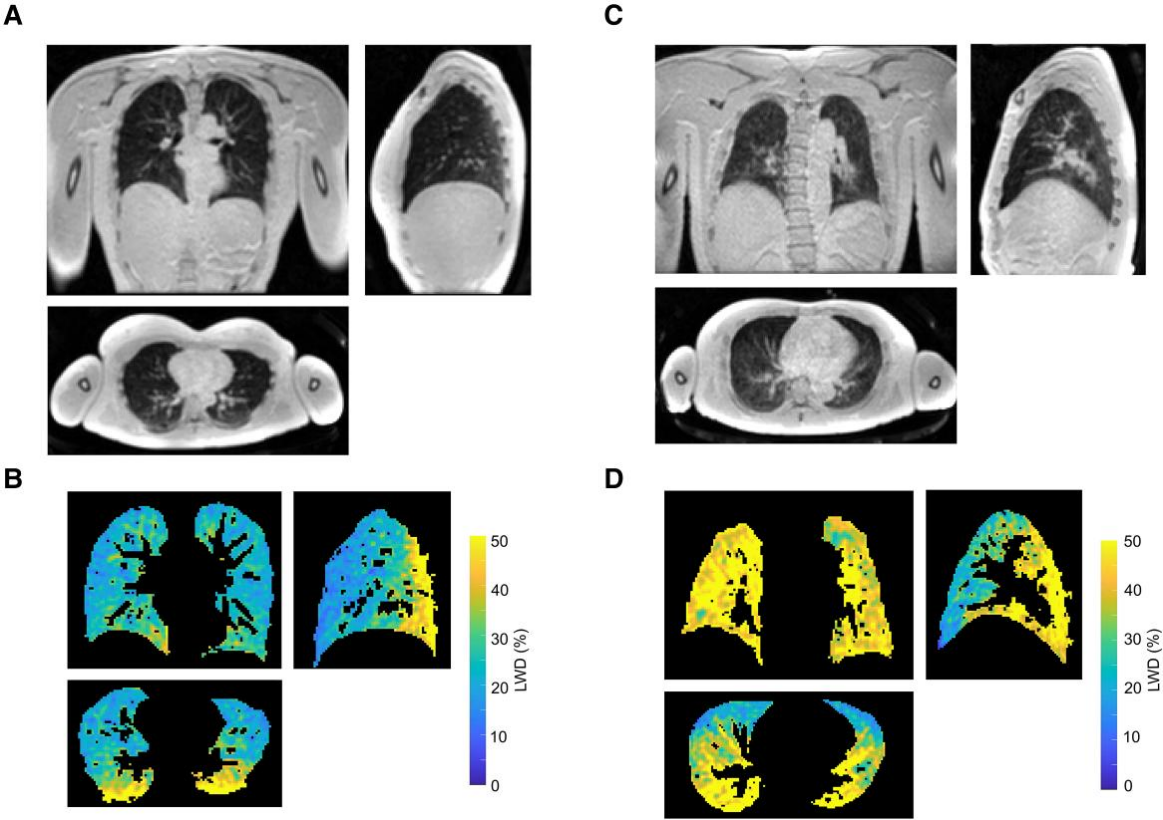
Illustrative lung images. Coronal, sagittal, and transverse slices from a 3D Cartesian lung water image in (*A*) control subject and (*C*) patient at risk of heart failure. LWD (%) in the lung parenchyma following image normalization and masking for the same slice locations shown in *A* (*B*) and *C* (*D*) showing globally increased LWD in the patient.

Univariable and multivariable analyses for LWD are shown in *Table 2*. In univariable linear regression analysis, LWD was associated with BSA, BMI, hypertension, RA area, LV mass, and GT (*Figure 4*). In a stepwise multivariable linear regression analysis including clinical and CMR variables associated with LWD, only BMI remained associated with LWD (model *R*^2^ = 0.32, *P* < 0.001). In a separate multivariable linear regression model including only CMR markers associated with LWD, RA area remained associated with LWD (model *R*^2^ = 0.05, *P* = 0.006) (*Table 3*). In subgroup analyses, LWD was higher in patients with hypertension (30.6 ± 5.0 vs. 27.5 ± 4.4%, *P* = 0.003) but did not differ according to sex or the presence of diabetes or coronary artery disease (*P* > 0.05 for all). Representative images showing LWD outputs are shown in *Figure 5*.

**Table 2:** Univariable and multivariable linear regression models of clinical and CMR variables associated with LWD

	Univariable model		Multivariable model, stepwise selection (global *R*^2^ = 0.32)	
	*R* ^2^	*P*-value	*t*	*P*-value
**Clinical**				
Age, years	0.019	0.07		
Male sex	0.003	0.52		
Body mass index, kg/m^2^	0.323	<0.001	8.9	<0.001
Body surface area, m^2^	0.121	<0.001	^a^	
Hypertension	0.051	0.003	^b^	
Diabetes	0.001	0.63		
Coronary artery disease	0.000	0.82		
NYHA functional class	0.001	0.69		
Systolic blood pressure, mmHg	0.018	0.09		
**CMR markers**				
RA area, cm^2^	0.045	0.006	^b^	
LA volume, mL	0.022	0.057		
LA volume indexed to BSA, mL/m^2^	0.001	0.71		
LV EDV, mL	0.018	0.09		
RV EDV, mL	0.009	0.23		
LV mass, g	0.035	0.01	^b^	
GT, mm	0.023	0.049	^c^	
LV ejection fraction, %	0.000	0.94		
GLS, %	0.009	0.26		
Presence of LGE	0.025	0.054		
Native T1, ms	0.002	0.63		
ECV, %	0.027	0.051		
LWV, mL	0.001	0.74		

Stepwise selection identified variables associated with LWD with *P* < 0.05 to enter and remain in the model.

NYHA, New York Heart Association; RA, right atrial; LA, left atrial; BSA, body surface area; LV, left ventricular; EDV, end-diastolic volume; RV, right ventricular; GLS, global longitudinal strain; GT, global LV wall thickness; LGE, late gadolinium enhancement; ECV, extracellular volume; LWV, lung water volume.

^a^Excluded from multivariable analysis due to collinearity with BMI.

^b^Assessed for inclusion in multivariable model but not retained.

^c^Excluded from multivariable analysis due to collinearity with LV mass.

**Table 3:** Univariable and multivariable linear regression models of CMR variables associated with LWD

	Univariable model		Multivariable model, stepwise selection (global *R*^2^ = 0.05)	
	*R* ^2^	*P*-value	*t*	*P*-value
**CMR markers**				
RA area, cm^2^	0.045	0.006	2.8	0.006
LA volume, mL	0.022	0.06		
LA volume indexed to BSA, mL/m^2^	0.001	0.71		
LV EDV, mL	0.018	0.09		
RV EDV, mL	0.009	0.23		
LV mass, g	0.035	0.01	^a^	
GT, mm	0.023	0.049	^b^	
LV ejection fraction, %	0.000	0.94		
GLS, %	0.009	0.26		
Presence of LGE	0.025	0.05		
Native T1, ms	0.002	0.63		
ECV, %	0.027	0.05		
LWV, mL	0.001	0.74		

Stepwise selection identified variables associated with LWD with *P* < 0.05 to enter and remain in the model.

RA, right atrial; LA, left atrial; BSA, body surface area; LV, left ventricular; EDV, end-diastolic volume; RV, right ventricular; GT, global LV wall thickness; GLS, global longitudinal strain; LGE, late gadolinium enhancement; ECV, extracellular volume; LWV, lung water volume.

^a^Assessed for inclusion in multivariable model but not retained.

^b^Excluded from multivariable analysis due to collinearity with LV mass.

### Variables associated with LWV

**Figure 6:**
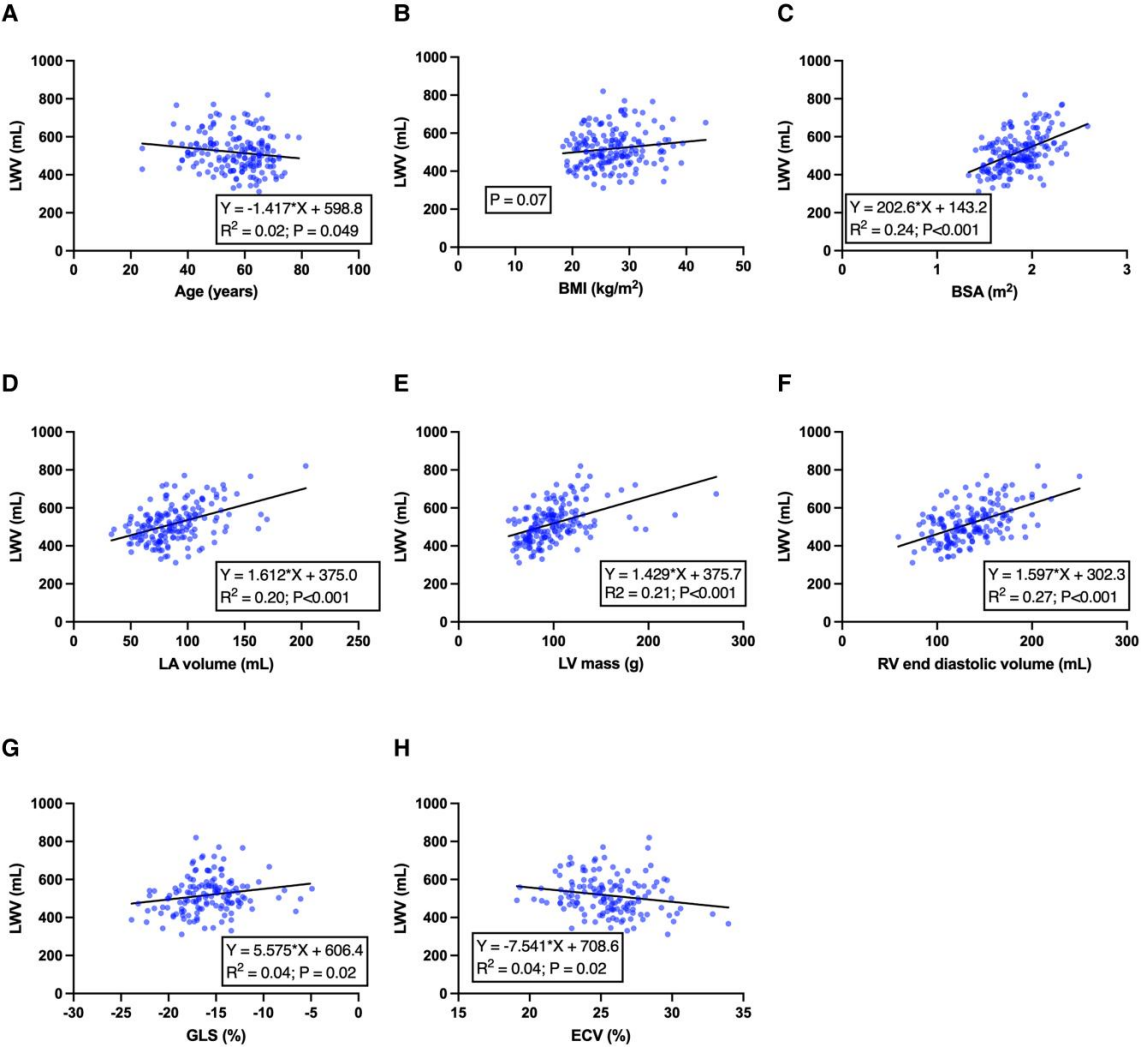
Scatter plots showing univariable relationships between LWV and clinical or routinely acquired CMR markers. LWV was significantly associated with age (*A*), BSA (*C*), LA volume (*D*), LV mass (*E*), RV end-diastolic volume (*F*), GLS (*G*), and ECV (*H*). The line of best fit is shown for each analysis.

Univariable and multivariable analyses for LWV are shown in *Table 4*. In univariable regression analysis, LWV was associated with age, male sex, BSA, LA volume, LV mass, GT, LV end-diastolic volume, RA area, right ventricular (RV) end-diastolic volume, and GLS (*Figure 6*). In a stepwise multivariable linear regression analysis including clinical and CMR variables, male sex, LA volume, and RV end-diastolic volume remained associated with LWV (global *R*^2^ = 0.40, *P* < 0.001). In subgroup analyses, LWV was higher in male subjects (549 ± 91 vs. 449 ± 72 mL, *P* < 0.001) but did not differ according to the presence of hypertension, diabetes, or coronary artery disease (*P* > 0.05 for all).

**Table 4:** Univariable and multivariable linear regression models of variables associated with LWV

	Univariable model		Multivariable model, stepwise selection (global *R*^2^ = 0.40)	
	*R* ^2^	*P*-value	*t*	*P*-value
Clinical				
Age, years	0.023	0.05	^a^	
Male sex	0.226	<0.001	4.9	<0.001
Body mass index, kg/m^2^	0.020	0.07		
Body surface area, m^2^	0.24	<0.001	^a^	
Hypertension	0.005	0.35		
Diabetes	0.009	0.21		
Coronary artery disease	0.007	0.29		
NYHA functional class	0.000	0.93		
Systolic blood pressure, mmHg	0.000	0.96		
CMR markers				
RA area, cm^2^	0.068	<0.001	^a^	
LA volume, mL	0.198	<0.001	3.9	<0.001
LA volume indexed to BSA, mL/m^2^	0.028	0.029	^b^	
LV EDV, mL	0.176	<0.001	^a^	
RV EDV, mL	0.275	<0.001	3.0	0.004
LV mass, g	0.208	<0.001	^a^	
GT, mm	0.104	<0.001	^c^	
LV ejection fraction, %	0.004	0.43		
GLS, %	0.037	0.02	^a^	
Presence of LGE	0.003	0.54		
Native T1, ms	0.011	0.21		
ECV, %	0.023	0.07		

Stepwise selection identified variables associated with LWV with *P* < 0.05 to enter and remain in the model.

NYHA, New York Heart Association; RA, right atrial; LA, left atrial; BSA, body surface area; LV, left ventricular; EDV, end-diastolic volume; RV, right ventricular; GLS, global longitudinal strain; LGE, late gadolinium enhancement; ECV, extracellular volume

^a^Assessed for inclusion in multivariable model but not retained.

^b^Excluded from multivariable analysis due to collinearity with LA volume.

^c^Excluded from multivariable analysis due to collinearity with LV mass.

### Lung volume and BMI

In univariable regression analysis, lung volume was noted to decrease with increasing BMI (*R*^2^ = 0.09, *P* < 0.001).

## Discussion

The main finding of this study is that LWD by CMR provides reasonable diagnostic performance for identifying patients at risk for heart failure and is poorly correlated with all other routinely acquired CMR markers of disease. Of the CMR markers of cardiac size, mass, function, and fibrosis tested, only RA area, LV mass, and global LV wall thickness were weakly correlated with LWD in univariable analyses. CMR-derived LWD has previously been shown to be strongly correlated with invasively measured left-sided filling pressures and B-type natriuretic peptide (BNP).^5^ Although pulmonary capillary wedge pressure can also be estimated using routine CMR markers using LA volume and LV mass, correlation with invasively derived filling pressures at right heart catheterization appears to be moderate at best.^16,17^ Therefore, CMR-derived LWD appears to provide important information regarding pulmonary congestion, possibly related to left-sided ventricular filling pressures, beyond what can currently be determined with conventional CMR markers.

In this study, LWD was higher in patients at risk for heart failure, compared with healthy controls. LWD measured using the HASTE technique was previously noted to be numerically higher in patients at risk compared with healthy controls, without reaching statistical significance.^5^ Our at-risk cohort was notably larger than the one presented in that study and also more diverse, consisting not only of patients with clinical cardiovascular risk factors but also including patients with a clinical history or CMR evidence of cardiomyopathy (without prior history of a heart failure event). These differences in cohort characteristics may have accounted for the increased LWD observed in our at-risk group. Furthermore, nearly all of the patients in the at-risk group had a history of hypertension, and this group demonstrated evidence of adverse cardiac remodelling, with increased LV mass and global LV wall thickness compared with the healthy controls, in addition to a higher LA volume. These structural changes hint at the presence of underlying LV diastolic dysfunction, which could not be confirmed as echocardiography was not performed in this study. Nonetheless, increased hydrostatic pressure in the pulmonary capillaries as a result of increased afterload in the setting of diastolic dysfunction and LV remodelling could possibly account for the increased LWD observed in the at-risk group.

A LWD cut-off value of 27.6% detected at-risk patients with an AUC of 0.70, indicating acceptable clinical performance.^18^ The specificity value of 73% is adequate and indicates that the majority of patients not at risk would be correctly classified. Conversely, the sensitivity value of 72% indicates that over a quarter of at-risk patients would be missed, which may be unacceptably high. The observed positive likelihood ratio value of 2.7 indicates that a positive test in most populations would increase the post-test probability by ∼15–20%.^19^ While this may be too small a change to be diagnostic, integration of LWD into a composite score assessing clinical risk factors and structural cardiac changes at CMR may improve diagnostic performance.

The weak relationship observed between LWD and conventional CMR markers can be explained by the underlying pathophysiology of pulmonary oedema. Pulmonary oedema occurs in heart failure when LA pressure exceeds a critical threshold, resulting in increased pulmonary capillary hydrostatic pressure.^1^ That threshold is variable, depending on the presence of a number of pulmonary adaptations, including reduced capillary filtration due to pulmonary vascular remodelling and endothelial dysfunction, enhanced alveolar fluid clearance, and increased lymphatic drainage.^20–23^ In some individuals, pulmonary capillary stress failure may occur due to pressure injury of the capillary wall and in the setting of pulmonary inflammation, resulting in alveolar flooding and oedema.^24^ Therefore, the relationship between LA pressure elevation and pulmonary congestion is not straightforward and likely substantially modified by the physiological regulation of the pulmonary vascular system, which is not well accounted for in conventional CMR. CMR-derived LWD therefore appears to provide complementary information to routinely acquired CMR markers. Further work is required to determine whether LWD-guided management strategies, including LWD-guided diuresis in patients with heart failure or integration of LWD into validated heart failure risk scores, can improve cardiovascular outcomes in heart failure beyond current algorithms.

In this study, LWD was most strongly associated with BMI. It is increasingly recognized that obesity is often associated with increased circulating blood volume and cardiac output, pulmonary venous, and subsequently arterial hypertension and, ultimately, with LV dysfunction and pulmonary oedema.^25,26^ Therefore, the relationship observed is in keeping with our current understanding of the pathophysiology of obesity. Furthermore, it is known that the compliance of the lungs, chest wall, and entire respiratory system is often reduced in obesity due, in part, to mediastinal and abdominal wall fat deposition.^27^ The resting volume of the lung, known as the functional residual capacity, is known to be reduced in proportion to the severity of obesity.^27^ Lung volume, calculated as the total voxel volume in the lung mask, showed an inverse correlation with BMI, in agreement with the literature. LWV trended higher with increasing BMI, although the correlation was not statistically significant. In the setting of unchanged or slightly increased LWV with increasing BMI and corresponding reduction in lung volume, LWD would be expected to rise as BMI increases, as observed. Furthermore, BMI showed stronger association with LWD than BSA in univariable regression analysis and was thus included in the multivariable model. Given the theoretical dependence of LWD on abdominal wall fat as discussed above, and due to the stronger dependence of BMI values on adipose tissue levels,^28^ this result is expected.

LWD more strongly correlated with RA area, compared with the left-sided chambers. This is an unexpected finding, since it is thought that LWD should more closely reflect left-sided filling pressures, rather than RV volume status, which is known to associate with RA area at echocardiography.^29–31^ RA dilatation may also be a marker of raised RA pressure, in the absence of significant tricuspid regurgitation,^32^ and therefore indirectly a marker of RV filling pressures. One possibility, therefore, is that LWD reflects a more global measure of fluid status, taking into account both left and RV preload. Nonetheless, the finding that LWD associates with RA size is novel and warrants further study.

The 3D Cartesian gradient-echo sequence used in this study to assess LWD has several advantages over the previously reported HASTE technique.^2,5,6,33^ The HASTE acquisitions use individual 2D slices, which provide incomplete coverage of the lungs. HASTE is a spin-echo sequence with inherent T2-weighing and thus potentially increased or decreased signal intensities without a change in water density. The HASTE sequence uses a highly asymmetric k-space (half-Fourier technique), which can result in image artefacts and cause spatial variations in signal that can confound water quantification. The use of a single user-selected liver region as reference tissue to convert MRI signals to water density with the HASTE approach will be prone to systematic errors due to coil shading effects that cause spatial heterogeneity in the MRI signals, independent of water density. Furthermore, the presence of liver disease, including fatty infiltration, iron deposition, or congestion, can contribute to inaccuracies in LWD measurement.

These limitations are largely overcome by the sequence used in this study. 3D full torso imaging provides complete coverage of the lungs and all surrounding solid tissues. The reference signal used is a composite of all background signals, including the liver, heart, and skeletal muscle, is user independent, and includes a signal normalization to correct for coil shading effects. A low flip angle (1°) eliminates T1 weighting.^2^ The automated segmentation enables removal of confounding tissues and conduit blood vessels. Furthermore, a built-in respiratory navigator enables automated end-expiration reconstruction with free-breathing acquisitions.

The LWD values obtained in this study were higher compared with previously reported LWD values using the HASTE sequence.^5,6^ This difference relates primarily to the differing methods used for background correction and normalization. In the previous studies, the liver was used as reference tissue and was assumed to have a water density of 70%. A similar correction was not applied in this study, in which the absolute water density in all of the surrounding tissues is acknowledged as unknown and assigned a reference value of 100%, and thus the LWD values are relative to the composite multi-tissue reference. Other reasons for systematic differences in LWD between the two techniques could also include differing amounts of T2, T2*, and T1 weighting.^2,5^

Apart from CMR, other non-invasive methods that permit direct, quantitative assessment of lung water include CT and nuclear medicine imaging with positron emission tomography (PET). PET enables determination of both intravascular and extravascular lung water (EVLW) through the use of two tracers, one which equilibrates with the total lung water pool and another that remains within the intravascular space. While lung water estimation by PET correlates strongly with gravimetric lung water weight and can identify patients with heart failure, this technique is costly, not widely available, and exposes the patient to ionizing radiation.^34–37^ CT density is expressed in Hounsfield units (HU), on a scale ranging from −1000 for air, 0 for water, and higher values for bone and metal. EVLW can be calculated from the mean lung density within a lung region of interest.^38^ Theoretical constraints for CT in evaluating lung water include dependence on the air volume in the lung and lack of signal specificity for water. Although animal studies have shown good correlation between lung density at CT and thermodilution and gravimetry techniques,^39^ results from human studies have been mixed.^40–43^ While CT is associated with an ionizing radiation burden, radiation doses are continually being reduced, and the technique is widely available, allows for fast imaging, and provides high spatial resolution.^1^ Recent advances, including an artificial intelligence quantitative CT algorithm that was able to identify subclinical pulmonary congestion in heart failure with preserved ejection fraction, suggest a larger role for CT lung water quantification in future.^40^

LWV was not able to discriminate between the healthy control group and patients at risk of heart failure in this cohort with a meaningful accuracy. This was likely due to the competing effects of higher LWD in the at-risk group and a trend towards lower lung volumes in that group. LWV showed strong association with markers of patient size, including sex and BSA. This is in keeping with the fact that EVLW measured using the transpulmonary thermodilution technique is known to be size dependent and is indexed to either actual or predicted body weight in clinical use.^44,45^ LWV indexed to BSA (LWVi) trended lower in the at-risk group, in part due to the inverse relationship between body size and lung volume. The between-group comparison, however, did not differ. LWVi is of potential clinical interest, as it may represent a non-invasive method to measure EVLW. Future work could compare LWVi with invasively measured EVLW in a more diverse cohort including patients with established heart failure, to determine its clinical significance. LA volume was independently associated with LWV in multivariable regression analysis, suggesting that LWV, in addition to LWD, may be associated with LV filling pressures. RV end-diastolic volume is known to be a marker of cardiac preload,^46^ which in turn is affected by volume status. The association between RV end-diastolic volume and LWV may therefore mechanistically result from increased fluid accumulation in the lungs in the setting of increased pulmonary vascular hydrostatic pressure due to an increased circulating blood volume.

### Limitations

In this study, we were not able to correlate the LWD results with BNP levels, a non-invasive marker of LV filling pressures, or invasively measured pulmonary capillary wedge pressure. The cohort assessed in this study included patients undergoing CMR for clinical indications or as part of other research studies at our institution, and these additional tests were therefore not included. Our cohort was overall relatively well and therefore only two patients met a combined outcome of hospitalization for heart failure or death over a median follow-up time of 20 months, limiting the ability to perform any meaningful survival analysis. Therefore, the clinical significance of our finding of higher LWD in patients at risk of heart failure is yet to be established and must be interpreted cautiously in that context. Future work should assess the independent prognostic significance of LWD derived from the 3D Cartesian gradient-echo sequence in patients at risk of and with established heart failure, with concurrent invasive measurement of LV filling pressure and BNP for further validation. LWD values in this study were calculated relative to a composite multi-tissue reference, consisting of the liver, heart, and skeletal muscle. Disease states affecting these tissues, including oedema in the setting of fluid overload, could lead to inaccuracies in those patient groups. LWD normal ranges would need to be established individually for each condition. Our cohort did not have established heart failure, and therefore, this issue was of negligible concern in the present study. Due to the retrospective and exploratory nature of the analysis, it was not possible to use separate derivation and validation cohorts to test the accuracy of LWD in identifying at-risk patients. This should be considered in future work, to better refine LWD cut-off values. Finally, the post-processing of LWD images was only semi-automated in this study, limiting ease of integration into routine clinical workflows. A fully automated offline LWD analysis tool has recently been developed, and data collection is underway in a heart failure cohort. Integration of this tool into the in-line workflow on MRI scanners from different vendors will facilitate widespread clinical adoption.

## Conclusion

LWD, as measured using an updated 3D Cartesian gradient-echo sequence at 1.5 T, overcomes a number of the limitations of the older HASTE sequences and is able to discriminate between healthy controls and patients at risk of heart failure. LWD is only weakly explained by routinely acquired CMR parameters.

## Consent

Ethics approval was obtained from the Singhealth Centralized Institutional Review Board in Singapore, and all participants provided written informed consent. The study was conducted in accordance with the principles of the Declaration of Helsinki.

## Data Availability

The data sets generated and analysed for the current study are not publicly available. Please contact the corresponding author for data requests.

## Lead author biography

Dr Nithin R. Iyer is an Australian cardiologist subspecialized in advanced cardiac imaging and PhD candidate at the University of Sydney, Australia. His thesis assesses the application of novel cardiovascular MRI techniques in heart failure. He is currently an associate consultant with the department of cardiology at Woodlands Health, Singapore.
